# Proteogenomic Analysis Reveals Proteins Involved in the First Step of Adipogenesis in Human Adipose-Derived Stem Cells

**DOI:** 10.1155/2021/3168428

**Published:** 2021-12-16

**Authors:** Bernardo Bonilauri, Amanda C. Camillo-Andrade, Marlon D. M. Santos, Juliana de S. da G. Fischer, Paulo C. Carvalho, Bruno Dallagiovanna

**Affiliations:** ^1^Laboratory of Basic Biology of Stem Cells (LABCET), Carlos Chagas Institute-Fiocruz/PR, Curitiba, Paraná 81350-010, Brazil; ^2^Laboratory for Structural and Computational Proteomics, Carlos Chagas Institute-Fiocruz/PR, Curitiba, Paraná 81350-010, Brazil

## Abstract

**Background:**

Obesity is characterized as a disease that directly affects the whole-body metabolism and is associated with excess fat mass and several related comorbidities. Dynamics of adipocyte hypertrophy and hyperplasia play an important role in health and disease, especially in obesity. Human adipose-derived stem cells (hASC) represent an important source for understanding the entire adipogenic differentiation process. However, little is known about the triggering step of adipogenesis in hASC. Here, we performed a proteogenomic approach for understanding the protein abundance alterations during the initiation of the adipogenic differentiation process.

**Methods:**

hASC were isolated from adipose tissue of three donors and were then characterized and expanded. Cells were cultured for 24 hours in adipogenic differentiation medium followed by protein extraction. We used shotgun proteomics to compare the proteomic profile of 24 h-adipogenic, differentiated, and undifferentiated hASC. We also used our previous next-generation sequencing data (RNA-seq) of the total and polysomal mRNA fractions of hASC to study posttranscriptional regulation during the initial steps of adipogenesis.

**Results:**

We identified 3420 proteins out of 48,336 peptides, of which 92 proteins were exclusively identified in undifferentiated hASC and 53 proteins were exclusively found in 24 h-differentiated cells. Using a stringent criterion, we identified 33 differentially abundant proteins when comparing 24 h-differentiated and undifferentiated hASC (14 upregulated and 19 downregulated, respectively). Among the upregulated proteins, we shortlisted several adipogenesis-related proteins. A combined analysis of the proteome and the transcriptome allowed the identification of positive correlation coefficients between proteins and mRNAs.

**Conclusions:**

These results demonstrate a specific proteome profile related to adipogenesis at the beginning (24 hours) of the differentiation process in hASC, which advances the understanding of human adipogenesis and obesity. Adipogenic differentiation is finely regulated at the transcriptional, posttranscriptional, and posttranslational levels.

## 1. Introduction

Obesity is the main risk factor for several diseases such as hypertension, diabetes, insulin resistance, dyslipidemia, heart diseases, and some cancer types; it is also related to increased overall mortality [1]. Human adipose tissue is a primary regulator of metabolism and energy balance and is composed of several cell types, including adipocytes, preadipocytes, mesenchymal stem cells, fibroblasts, macrophages, and endothelial and blood cells [2, 3]. The pathophysiology of obesity is directly linked to increased adipocyte hypertrophy, adipocyte hyperplasia, or both in adipose tissue. Therefore, adipocyte size and the counterbalance between adipocyte loss and their formation through differentiation of preadipocytes are critical for human health. Therefore, adipogenesis plays a key role in the development of obesity, being the hormonal and nutritional status critical for the maintenance and development of body fat tissue [4]. Over the past two decades, adipogenesis has been extensively studied at the cellular and molecular levels, both *in vitro* and *in vivo* [4–8].

Adipose tissue is an abundant source of human adipose-derived mesenchymal stem cells (hASC), which have the ability to differentiate into adipocytes, osteoblasts, and chondrocytes [2, 9]. The adipogenic differentiation process is separated into two stages: the commitment of hASC into preadipocytes and the terminal differentiation of preadipocytes into mature adipocytes [10, 11]. Both phases are highly regulated at the transcriptional, posttranscriptional, translational, and posttranslational levels [12–14]. However, few studies have focused on molecular regulation at the earliest steps in hASC differentiation into preadipocytes [15, 16]. Thus, understanding how hASC become committed to preadipocytes and adipocyte lineages is a principal concern in the study of adipose tissue homeostasis and development.

Different techniques have been used to understand molecular changes at a global level (*e.g*., transcriptomics and proteomics) to better understand the initial stage of adipogenesis [17–19]. In a previous study, we used ribosome profiling (Ribo-seq) to identify the translational regulation of mRNAs after 72 hours of hASC adipogenesis. We observed a significant reduction in cell size and migration, in addition to a reduction in protein synthesis after induction [20]. Similarly, we used total fraction and polysome profiling followed by RNA-seq to show the downregulation of cell cycle- and proliferation-related genes after 24 hours of induction [21]. Using these distinct high-throughput sequencing techniques of different cell fractions (i.e., bulk RNA-seq and polysomal and ribosome-associated mRNAs) may reflect a better understanding of the posttranscriptional and translational regulation of RNAs during cell differentiation, making possible a further comparison of gene expression at the RNA level with proteomics data (i.e., protein level). However, as far as we know, only one study discloses proteomic results from the early stages of human adipogenesis, focusing only on the expression of transcription factors [22, 23].

With this as a motivation, we performed a proteogenomic analysis of the early steps of adipogenesis (24 hours) in hASC. Our findings include the identification of proteins regulated at the beginning of the adipogenic differentiation process in hASC. This gene expression regulation is exerted at multiple levels, showing that human adipogenesis is a complex process and that combined strategies must be used for a better understanding.

## 2. Material and Methods

### 2.1. Ethical Statement

Tissue collection and cell isolation were performed after donors provided informed consent in accordance with guidelines for research involving human subjects and with the approval of the Ethics Committee of the Oswaldo Cruz Foundation, Brazil (approval number CAAE: 48374715.8.0000.5248). Human adipose-derived stem cells (hASC) were obtained from adipose tissue from lipoaspirate samples from three female donors (biological replicates) with a mean (±SD) age of 38 ± 12.75 and BMI of 27.06 ± 1.88 (Table 1). We randomly selected the donors since no differences in morphology, immunophenotype characteristics, proliferative rates, and differentiation potential between hASC isolated from young and old subjects were demonstrated [24].

### 2.2. Isolation, Cell Culture, and Characterization

Cell isolation was performed as previously described [25]. Briefly, 200 mL of adipose tissue was washed with phosphate-buffered saline (PBS) (Gibco, Invitrogen), after which digestion was carried out using 1 mg/mL type I collagenase (Gibco) for 30 min at 37°C, 5% CO_2_ under constant shaking. Next, the cell suspension was treated with hemolysis buffer and filtered through a 100 *μ*m a 40 *μ*m mesh filter (BD Biosciences). Finally, the cells obtained were washed and plated at a density of 1 × 10^5^ cells/cm^2^ in T75 culture flasks in DMEM supplemented with 10% FBS, penicillin (100 units/mL), and streptomycin (100 *μ*g/mL) in humid incubator at 37°C and 5% CO_2_. The culture medium was changed twice a week, and all experiments were performed with cell cultures at passages four to six. For adipogenic induction, hASC were cultured for 24 hours in hMSC Adipogenic Differentiation Medium (hMSC Adipogenic Differentiation, BulletKit, Lonza). Cell characterization was performed according to the minimal criteria established by the International Society of Cellular Therapy [26], being the flow cytometry analysis conducted as previously described [27] (Supplementary Figure S1).

### 2.3. Sample Preparation and Quantification

Proteins were extracted with RapiGest™ detergent at a concentration of 0.1%. Protein concentrations were quantified using the fluorimetric assay on the Qubit platform (Invitrogen), following the manufacturer's instructions. One hundred micrograms of protein from each sample was reduced with dithiothreitol (DTT) (final concentration of 10 mM) for 30 minutes at 60°C. After incubation at room temperature, the samples were alkylated with iodoacetamide (final concentration of 30 mM) for 25 minutes at room temperature at dark and finally digested with trypsin in the proportion of 1/50 (E/S) for 20 hours.

The enzymatic reaction was interrupted by adding trifluoroacetic (0.4% *v*/*v* final). Peptides were successively quantified using the fluorometric test on Qubit 2.0® (Invitrogen) according to the manufacturer's recommendations. Each sample was desalted and concentrated using Stage-Tips (STop and Go-Extraction TIPs) according to Rappsilber and colleagues [28].

### 2.4. Mass Spectrometry Analysis

The peptide mixture was suspended in 0.1% formic acid and analyzed as follows. An UltiMate 3000 Basic Automated System (Thermo Fisher®) was set up and connected online with a Fusion Lumos Orbitrap mass spectrometer (Thermo Fisher®). The peptide mixture was chromatographically separated on a column (15 cm in length with an internal diameter of 75 *μ*m) packed in-house with ReproSil-Pur C18-AQ 3 *μ*m resin (Dr. Maisch GmbH HPLC) with a flow rate of 250 nL/min of 5% to 50% ACN in 0.1% formic acid on a 140 min gradient. The Fusion Lumos Orbitrap was placed in data-dependent acquisition (DDA) mode to automatically turn between full-scan MS and MS/MS acquisition with 40 s dynamic exclusion. Survey scans (200–1500 m/z) were acquired in the Orbitrap system with a resolution of 120,000 at *m*/*z* 200. The most intense ions captured in a 2 s cycle time were chosen, excluding those which were unassigned or had a 1+ charge state. The selected ions were then isolated in sequence and fragmented using HCD (higher-energy collisional dissociation) with a normalized collision energy of 30. The fragment ions were analyzed with a resolution of 15,000 at 200 m/z. The general mass spectrometric conditions were as follows: 2.5 kV spray voltage, no sheath or auxiliary gas flow, heated capillary temperature of 250°C, predictive automatic gain control (AGC) enabled, and an S-lens RF level of 40%. Mass spectrometer scan functions and nLC solvent gradients were regulated using the Xcalibur 4.1 data system (Thermo Fisher®).

### 2.5. Peptide Spectrum Matching (PSM)

Data analysis was performed with the PatternLab for Proteomics 5 software, which is freely available at http://www.patternlabforproteomics.org. Data analysis was performed according to the software's protocol [29]. Briefly, the sequences from *Homo sapiens* were downloaded on December 17, 2020, from the Swiss-Prot database, and a target decoy database was generated to include a reversed version of each sequence plus those from 104 common mass spectrometry contaminants. The Comet 2019.01 rev. 5 search engine was used to identify the mass spectra [30]. The search parameters considered were: fully and semitryptic peptide candidates with masses between 550 and 5500 da, up to two missed cleavages, 40 ppm for precursor mass, and bins of 0.02 m*/z* for MS/MS. The modifications were carbamidomethylation of cysteine as fixed and oxidation of methionine as a variable.

### 2.6. Validation of PSMs

The validity of the PSMs was assessed using Search Engine Processor (SEPro) [31]. First, the identifications were grouped by charge state (2+ and ≥3+) and then by tryptic status, resulting in four distinct subgroups. For each group, the XCorr, DeltaCN, DeltaPPM, and Peak Match values were used to generate a Bayesian discriminator. Next, the identifications were sorted in nondecreasing order according to the discriminator score. Finally, the cutoff score accepted a false discovery rate (FDR) of 1% at the peptide level based on the number of decoys [32]. This process was independently performed on each data subset, resulting in an FDR independent of the charge state or tryptic status. Moreover, a minimum sequence length of six amino acid residues was imposed, as was and a protein score greater than two were imposed. Lastly, identifications deviating by more than 10 ppm from the theoretical mass were discarded. This last filter decreased the rate of FDRs, now at the protein level, to less than 1% of all search results [33].

### 2.7. Proteomic Data Analysis

Our experimental design considered three biological replicates for each biological condition (*i.e*., adipogenic induction (ADI) and undifferentiated cells (CT)), with two technical replicates. Quantitation was performed using Extracted Ion Chromatograms (XICs) normalized according to the total ion current (Supplementary Table 1). Differentially abundant proteins were listed by using PatternLab's TFold module to compare ADI versus CT [34].

### 2.8. Transcriptomic Data Analysis

Our previous RNA-seq data was used to perform transcriptomic analysis [25]. Briefly, bioinformatics analyses for mapping and counting were performed with the *rsubread* package with the new human genome version (GRCh38). Parameters were set for unique mapping reads. To determine the differential expression between undifferentiated hASC (CT) and 24 h-differentiated hASC (ADI), only genes with a count of more than 1 per million in at least three conditions were considered. To identify differentially expressed genes (DEGs), we used the *edgeR* Bioconductor package [35]. DEGs were selected using a stringent analysis using a false discovery rate (FDR) threshold of ≤0.05 (5%) and log_2_ fold change (log_2_FC) ≥ 1.5 or ≤-1.5. All statistical analyses were conducted in R.

Gene Ontology (GO) analysis of the identified proteins was performed with the Database for Annotation, Visualization, and Integrated Discovery (DAVID) [36], using a cutoff criterion of *P* ≤ 0.05 for “biological processes.” Predicted protein-protein interaction networks were performed with STRING [37] using default parameters, and the network was created with Cytoscape (v.3.5.0).

## 3. Results

### 3.1. Study and Sample Overview

**Figure 1:**
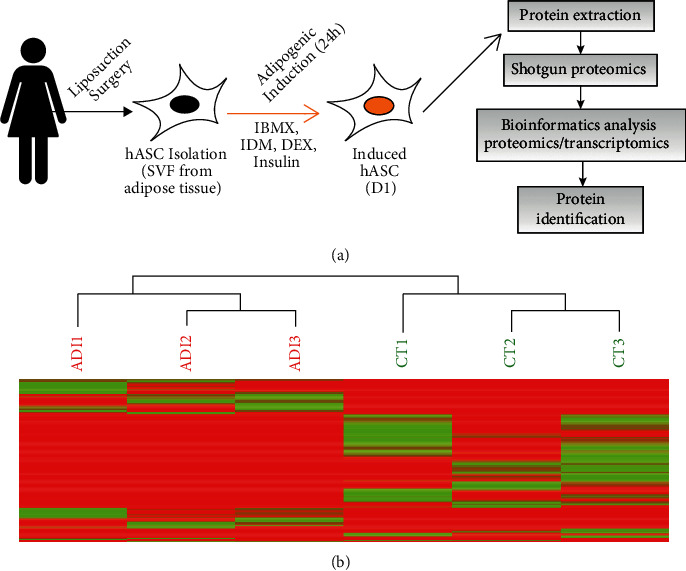
Triggering adipogenesis in hASC. (a) Schematic illustration of adipose tissue collection following by hASC isolation and *in vitro* adipogenic differentiation process with standard cocktail. (b) The clustergram was generated by applying hierarchical clustering to the ADI and CT proteomic profiles. The red and green indicate ADI and CT samples, respectively. Each line encodes the NIAF quantitation for a peptide; the encoding ranges from red for low-intensity NIAF quantitation to green for high-intensity NIAF quantitation.

We isolated hASC from human adipose tissue obtained after liposuction surgery of three female subjects, followed by cell characterization and expansion, as previously described [21, 27]. Afterward, hASC were induced to adipogenic differentiation by culturing with a differentiation induction medium (*i.e*., adipogenic cocktail (ADI)) for 24 hours. Noninduced cells were cultured with control-supplemented medium (CT). Cells were harvested, and proteins were immediately extracted (see Material and Methods). Next, we performed a shotgun proteomic analysis using the Fusion Lumos Orbitrap mass spectrometer followed by bioinformatic analysis (Figure 1(a)). We used PatternLab's Clustergram module to perform unsupervised clustering of the proteomic profiles of ADI and CT samples; the results show a clear stratification between the two experimental conditions (Figure 1(b)). Finally, the assessment of the chromatographic reproducibility of technical replicates was performed using RawVegetable [38] software (Supplementary Figure S2).

### 3.2. Uniquely Identified Proteins in 24 h-Differentiated (ADI) versus Undifferentiated (CT) hASC

**Figure 2:**
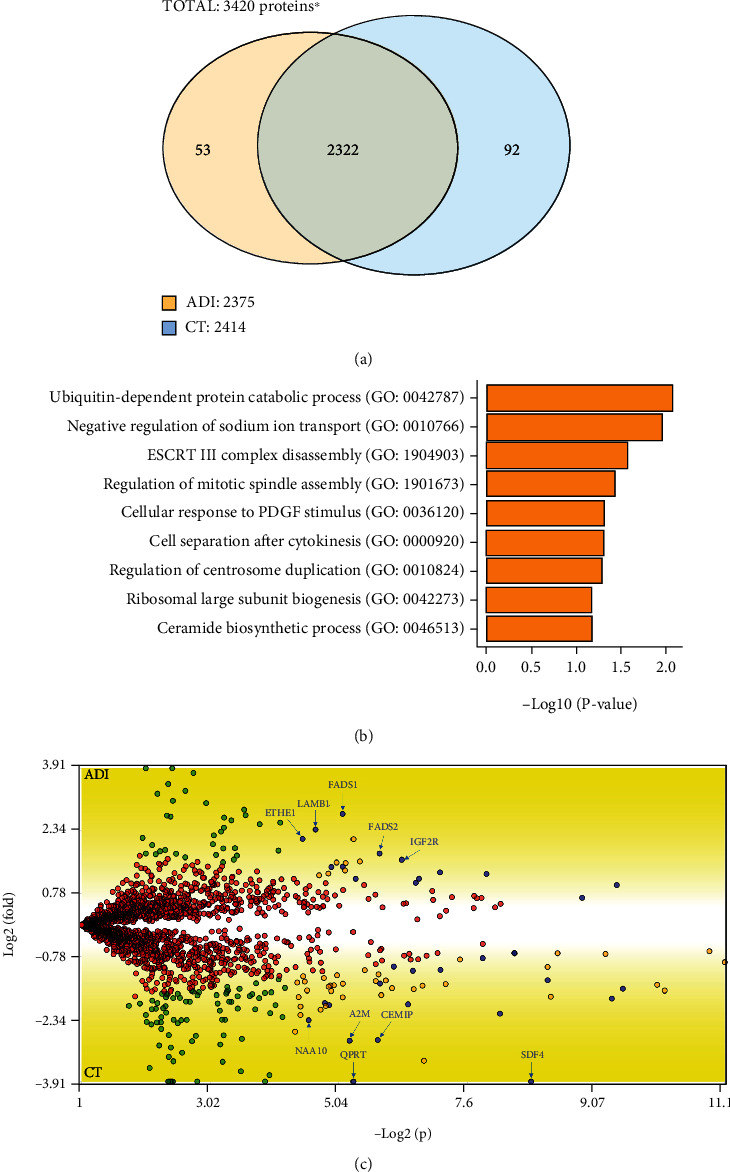
Proteins identified in 24 h-differentiated and undifferentiated hASC. (a) A total of 3420 proteins were identified. The Venn diagram shows that 2322 proteins are common to 24 h-differentiated (ADI) and undifferentiated hASC (CT) conditions. Cells exposed to 24 hours of adipogenic cocktail have 53 exclusive proteins. Undifferentiated hASC have 92 exclusive proteins. ^∗^The Venn diagram represents the number of proteins that satisfy a minimum of 2 replicates. (b) Gene Ontology analysis of the 53 uniquely identified proteins after adipogenic induction. Only biological processes with *P* ≤ 0.05 are shown. (c) Differently abundant proteins identified in hASC exposed to 24 hours of adipogenic differentiation. Blue dots represent the 33 proteins that satisfy our stringent criteria. Red dots represent proteins with no satisfying fold change cutoff and established FDR. Green dots represent proteins that satisfy only the fold change cutoff. Orange dots represent proteins that satisfy both criteria but received very low quantitative values. The five proteins with the highest and lowest abundance are named.

To understand the changes in the proteome of hASC after the first day of adipogenic differentiation, we performed a shotgun proteomic approach followed by a proteomics analysis using the *PatternLab for Proteomics* protocol [10]. We were able to identify 53 exclusive proteins in two or more biological replicates in the ADI condition and 92 exclusive proteins in the CT condition, with 2322 proteins common in both conditions (Figure 2(a)). All exclusive proteins were manually curated in the scientific literature (Supplementary Table 1). Next, we performed a GO analysis of exclusive proteins from the ADI condition and found biological processes related to protein catabolism, negative regulation of sodium ion transport, ESCRT III complex, disassembly, regulation of mitotic spindle assembly, ribosomal large subunit biogenesis, and ceramide biosynthetic process (Figure 2(b)). We observed biological process pathways related to angiogenesis, mitochondrial translation, and chloride transmembrane transport for the exclusive proteins from CT condition. In addition, pathways related to cell-cell adhesion, translation initiation, and cotranslational membrane targeting were shown for common proteins (Supplementary Figure S3). Several ADI proteins have at least one report related to adipogenesis, adipocytes, or adipose tissue. This is the case of the ASAP1 protein, which regulates cytoskeletal dynamics and intracellular vesicle trafficking [39]. Another interesting example is the cochaperone FKBP5, which represses the Akt-p38 kinase pathway, inhibiting the glucocorticoid receptor-*α* (GR*α*) while also stimulating PPAR*γ* [40–42].

We also found exclusively CT proteins with at least one report related to differentiation, mesenchymal stem cells, and adipogenesis. We highlight the cellular retinoic acid-binding protein II (CRABP2), which mediates the accessibility of retinoic acid (RA) to different receptors (RARs). Previous reports show that RA inhibits adipocyte differentiation when administered during the early steps of differentiation [43, 44].

### 3.3. Differentially Abundant Proteins after Adipogenic Triggering

We performed a differential analysis to visualize changes in protein abundance between ADI and CT using the PatternLab T-Fold analysis under stringent criteria (see Material and Methods). The analysis showed that 33 proteins had significantly different abundance levels between treatments, with 14 proteins upregulated and 19 downregulated after 24 hours of adipogenic differentiation (Figure 2(c), Supplementary Table 2). The upregulated proteins were related to pathways linked to the oxidation-reduction process, alpha-linolenic acid metabolic process, unsaturated fatty acid biosynthetic process, cell-cell adhesion, response to drugs, and organic cyclic compounds, cell spreading, and response to insulin (Supplementary Figure S4A). On the other hand, the downregulated proteins related to pathways involving response to amino acid stimulus, collagen catabolic process, protein heterotrimerization, positive regulation of cell migration, extracellular matrix organization, endochondral ossification, endodermal cell differentiation, iron-ion homeostasis, and collagen fibril organization were identified (Supplementary Figure S4B).

### 3.4. Hidden Proteins Identified in Proteomics

**Figure 3:**
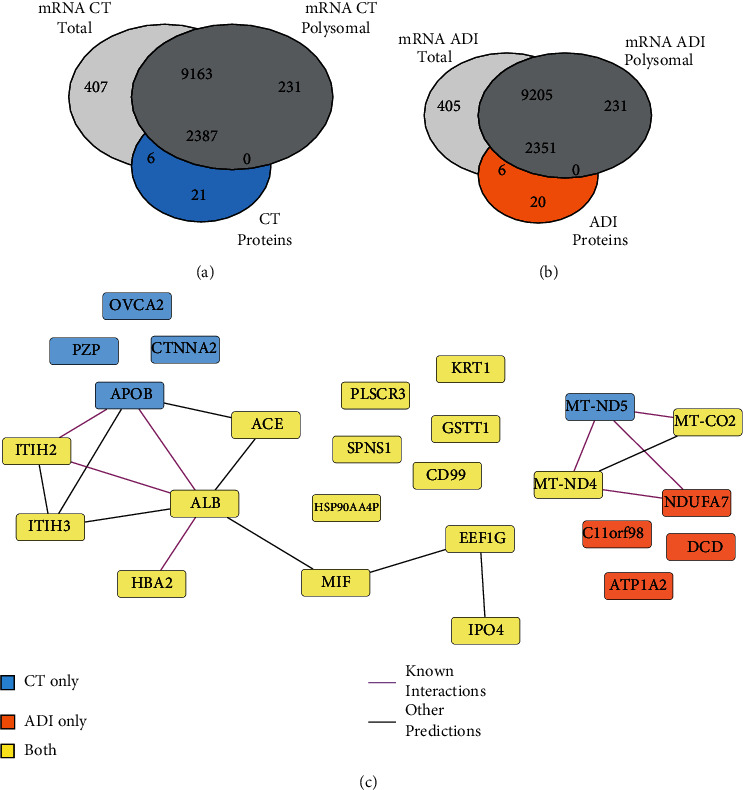
Unseen genes in the transcriptome are revealed by proteomics. (a) Venn diagram comparing all proteins and total mRNAs identified in the CT (left Venn diagram) and ADI (right Venn diagram) conditions. (b) Venn diagrams comparing all proteins and polysomal mRNAs identified in the CT (left Venn diagram) and ADI (right Venn diagram) conditions. Boxes show hidden proteins detected exclusively in the CT (5 proteins) and ADI (4 proteins) conditions. (c) Protein-protein interaction network demonstrating the identified hidden proteins. CT represents undifferentiated hASC, ADI represents 24 h-differentiated cells, and BOTH represents total and polysomal fractions.

To study protein expression regulation, we verified the presence of all identified proteins in the transcriptomics data. We found that approximately 99.13% of the identified ADI (2355 of 2375) and CT (2393 of 2414) proteins were assigned to the corresponding transcripts detected by RNA-seq in the total fraction (Figure 3(a)). Similarly, approximately 98.85% of the identified ADI (2350 of 2375) and CT (2387 of 2414) proteins were assigned to the corresponding transcripts detected by RNA-seq in the polysomal fraction (Figure 3(b)). We found 16 common proteins lacking an mRNA cognate in ADI and CT conditions across the total and polysomal fractions (Figure 3(c)). In the CT condition, only five proteins were missing from RNA-seq data across both fractions (*i.e.*, CTNNA2, OVCA2, MT-ND5, APOB, and PZP) (Figure 3(c)). Little is known about these proteins, particularly regarding their function and mechanisms in stem cells. On the other hand, in the ADI condition, only four proteins are missing from RNA-seq data across both fractions (*i.e.*, C11orf98, DCD, ATP1A2, and NDUFA7) (Figure 3(c)). Little is known about the role of these proteins in adipogenesis and stem cell biology.

Taken together, these results shed light on these hidden proteins, which are missing from the RNA-seq data, enabling new hypotheses regarding their participation in the self-renewal and differentiation processes of hASC.

### 3.5. Comparison of Proteomics and Transcriptomics Data

To gain more in-depth insights, we used our previous next-generation sequencing data (RNA-seq) of the total and polysomal fractions of hASC submitted to 24 hours of adipogenic differentiation to understand the different levels of gene expression regulation [25].

**Figure 4:**
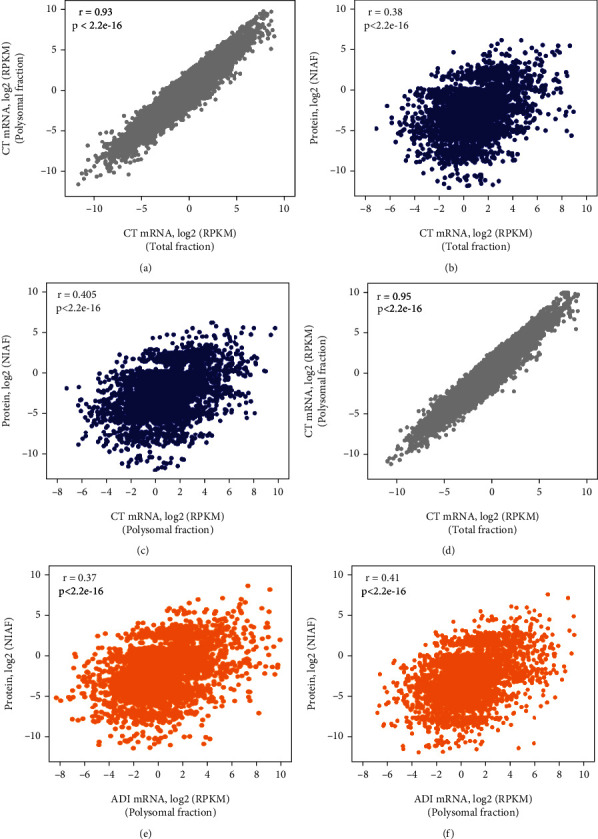
Relationship of identified proteins and mRNAs in control and induced hASC. (a) Correlation coefficient of CT mRNAs from the total and polysomal fractions. (b, c) Proteins versus CT mRNAs from the total and polysomal fractions, respectively. (d) Correlation coefficient of ADI mRNAs from the total and polysomal fractions. (e, f) Proteins versus ADI mRNAs from the total and polysomal fractions, respectively.

To understand the relationship between mRNA and protein levels, we calculated Spearman's correlation coefficient of normalized mRNA counts and normalized protein intensity of the induced (ADI) and control cells (CT) (Supplementary Table 3). First, we compared RNA-seq data from the total and polysomal fractions. We observed a high positive correlation coefficient (Figures 4(a) and 4(d)). Furthermore, a significant positive correlation between mRNA and protein was observed when comparing protein levels with mRNAs from total RNA (CT: 0.38, *P* < 2.2*e* − 16; ADI: 0.37, *P* < 2.2*e* − 16) and polysomal RNA fractions (CT: 0.40, *P* < 2.2*e* − 16; ADI: 0.41, *P* < 2.2*e* − 16). The correlation with the polysomal fraction is slightly higher for both conditions (Figure 4).

**Figure 5:**
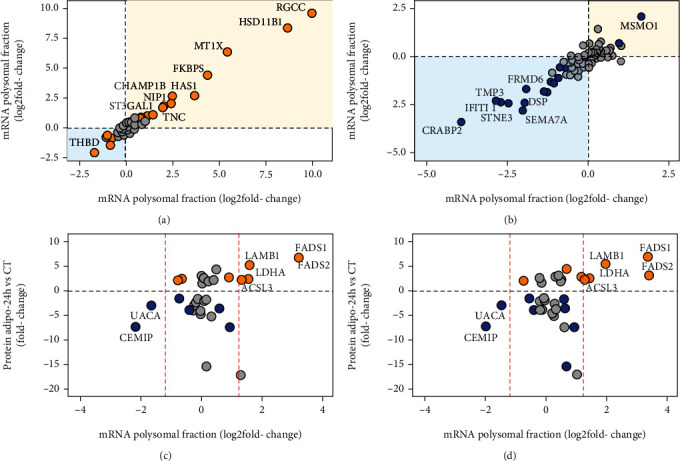
Gene expression regulation during the first step of human adipogenesis. (a) Scatterplot comparing RNA-seq from the total and polysomal fractions of hASC submitted to adipogenesis (24 hours), showing 53 proteins uniquely identified in 24 h-differentiated cells. (b) The same analysis with the 92 proteins uniquely identified in undifferentiated hASC. Orange and blue dots represent genes with FDR ≤ 5%. The named dots represent DEGs in the total and polysomal fractions. Orange and blue boxes represent adipogenesis (24 hours) and undifferentiated hASC, respectively. The differentially abundant proteins (up- and downregulated) were compared with the mRNA expression of the (c) total and (d) polysomal fraction. Orange and blue dots represent genes with FDR ≤ 5% in the ADI and CT conditions, respectively. Dashed red lines represent the cutoff criteria for fold change in the RNA-seq data.

To analyze the relationship between mRNA and protein levels in greater detail, we first observed differential mRNA expression in both RNA fractions. We determined a cutoff criterion for DEGs of fold change (log_2_) ≥ 1.5 or ≤-1.5 and FDR ≤ 5%. We identified 407 upregulated and 580 downregulated DEGs in the total RNA fraction, and 481 upregulated and 585 downregulated DEGs in the polysomal RNA fraction (Supplementary Table 4). We then examined the mRNA expression of the unique and differentially abundant proteins identified in our proteomic analysis.  Figure 5(a) shows the mRNA profile of the 53 exclusive ADI proteins in the RNA-seq data. Most mRNAs in total and polysomal fractions followed the upregulation expression after adipogenic induction (Figure 5(a); orange box), including nine DEGs. However, some mRNAs appear to be downregulated (Figure 5(a); blue box), which may indicate a specific posttranscriptional regulation of these transcripts. The differentially expressed mRNA THBD (thrombomodulin), which is involved in development, adipogenesis, and lipid metabolism, is one such example. THBD mRNA was shown to change during adipogenesis, while it is significantly increased after 72 hours of adipogenic differentiation [45–47]. Here, we demonstrate the presence of the THBD protein in cells undergoing early differentiation and downregulation of THBD mRNA in the total and polysomal fractions after this step.

Figure 5(b) shows the mRNA profile of the 92 exclusive CT proteins in the RNA-seq data. As seen above, the same pattern emerges for the CT proteins, with most mRNAs in total and polysomal fraction downregulated after adipogenic induction (Figure 5(b); blue box), including seven DEGs. Meanwhile, some mRNAs appear upregulated (Figure 5(b); orange box). Curiously, the mRNA MSMO1 appears to be differentially upregulated in the total and polysomal fractions, which may indicate a distinct regulation of the transcript. In a study of adipogenesis in 3T3-L1 preadipocytes, Xin et al. used RNA-seq to demonstrate that the expression of MSMO1 was downregulated after 13 days of induction. The knockdown of MSMO1 stimulated the differentiation and upregulation of the expression of adipogenic marker genes, while MSMO1 overexpression had the opposite effect [48].

Interestingly, only seven differentially abundant proteins (five upregulated and two downregulated) are differentially expressed in transcriptomic data, both in the total and polysomal fraction, respectively (Figures 5(c) and 5(d)). With this result, we concluded that of the 14 upregulated proteins, nine (64%) cognate mRNAs did not undergo significant changes. Moreover, of the 19 downregulated proteins, 17 (89%) cognate mRNAs did not undergo significant changes in their expression, which may indicate a posttranscriptional regulation of these transcripts and turnover control of these downregulated proteins.

## 4. Discussion

### 4.1. Differentially Expressed Proteins and mRNAs

In this study, we used a proteomic approach to identify proteins that are differentially expressed at the onset of adipogenic differentiation of hASC. This approach complements previous studies using RNA-seq analysis and allowed us to find new potential early markers of differentiation. FKBP5 and ASAP1 proteins appear to be interesting differentiation-related markers.

FKBP5 is an adipocyte differentiation marker in 3T3-L1 cells, and knockdown of the protein leads to a reduction in lipid accumulation and the expression of adipogenic-related genes (including PPAR*γ*) *in vitro* and *in vivo* [40, 42]. Dexamethasone exposure directly increases the FKBP5 gene and protein expression in human subcutaneous and omental adipose tissues [41]. Our results demonstrated the expression of FKBP5 at the very beginning of the adipogenic differentiation process in hASC, possibly regulated by dexamethasone exposure.

Another interesting protein is ASAP1, a regulator of cytoskeletal dynamics. Loss of ASAP1 causes growth retardation, delayed ossification, and reduced adipogenesis *in vivo*, suggesting an essential role for this protein in mesenchymal differentiation [39].

We also found that CRABP2 was only present in nondifferentiated cells and tightly downregulated at the beginning of differentiation. The effect of RA is related to the activation of RARs, which is mediated by CRABP2. However, when preadipocytes are exposed to three components of the adipogenic cocktail (*i.e*., insulin, IBMX, and DEX), the expression of CRABP2 is repressed, and the RA effects are inhibited [43, 44]. We previously demonstrated the downregulated expression of the mRNA CRABP2 after 24 hours of adipogenic and osteogenic differentiation of hASC, reinforcing its importance in the maintenance of the undifferentiated state [25].

When comparing our proteomic data with previous gene expression profiles of differentiated hASC, we were not able to find the related mRNAs for a small group of proteins (Figure 3). However, a closer analysis of the proteins revealed that many of them were serum proteins, probably contaminants from the culture media (*e.g.*, ALB) (Supplementary Figure S5). This shows that, although they were thoroughly washed, the cells retained proteins from the serum present during cultivation. Another group of proteins was of mitochondrial origin (*e.g.*, MT-ND4 and MT-CO2). Most of these were coded in the mitochondrial genome and are probably the result of a bias of the RNA-seq assay.

We identified proteins with differential abundance after 24 hours of adipogenic differentiation. The mRNAs corresponding to the most upregulated proteins were also differentially expressed in the total and polysomal fractions. We highlight *Δ*-5 Fatty Acid Desaturase (FADS1) and *Δ*-6 Fatty Acid Desaturase (FADS2), proteins that regulate the biosynthesis of polyunsaturated fatty acids (PUFA). Altered desaturase activity is an important issue in adipose tissue, body weight, and glucose uptake. FADS1 and FADS2 are expressed in adipocytes and have a functional pathway that can be regulated by PUFA. Thus, both eicosapentaenoic acid (EPA) and arachidonic acid (AA) reduced the expression of FADS1 and FADS2, while alpha-linoleic acid (ALA) and linoleic acid (LA) did not [49]. Genetic polymorphisms in FADS genes are associated with changes in fatty acid metabolism (*e.g*., concentration), as well as overweight, obesity, metabolic syndrome, and cognition functions [50–53]. Furthermore, knockout- (KO-) Fads2 mice develop obesity resistance and impaired lipogenesis [54]. We previously demonstrated the expression of FADS2 mRNA in total and polysomal fractions after 24 hours of adipogenesis in hASC [25], while its expression at the protein level is demonstrated in this study. Similarly, the ACSL3 protein is a member of the long-chain acyl-CoA synthetase family, which plays an important role in fatty acid metabolism. The protein is located in the endoplasmic reticulum (ER), from where it is effectively translocated to lipid droplets when lipid synthesis is stimulated. Its overexpression promotes an increase in the triglyceride content of lipid droplets. Therefore, ACSL3 plays a key role in adipocyte differentiation [55, 56].

Another interesting protein is the laminin subunit beta 1 (LAMB1), which has functions related to the extracellular matrix (ECM). Recently, high levels of LAMB1 were observed in visceral adipose tissue and during *in vitro* adipogenesis. Obesity alters the gene expression profile of ECM genes in adipose tissue (LAMB1 was upregulated in visceral and subcutaneous adipose tissue) [57–60]. The lactate dehydrogenase A protein (LDHA) catalyzes the conversion of pyruvate to lactate in the glycolytic metabolism. Although insulin stimulates glycolysis, the lactate production of adipocytes not only depends on glucose availability and uptake but is also maintained during insulin resistance. This indicates that lactate production is an important aspect of adipocyte and adipose tissue metabolism [61]. Here, we demonstrate the early expression of LDHA in adipogenic differentiation, though the mechanism and influence of this protein during the differentiation process remain unknown.

### 4.2. Posttranscriptional Regulation of Differential Abundant Proteins

We are interested in studying the posttranscriptional regulation of the hASC differentiation process. To do so, we used different global approaches to determine the gene expression profile in the transcriptome, translatome, and proteome on the first day of adipogenic differentiation. We found a strong correlation between the transcriptome and the translatome, corroborating our group's previous findings [14]. We have observed that regulation of mRNA abundance is predominant at the initial steps of adipogenesis, with translational regulation increasing on the last days of differentiation [14, 20, 21]. A positive correlation was also observed between mRNA and protein expression data. Despite the positive correlation between mRNAs and proteins, the differences may be related to the different half-lives of the proteins and/or mRNAs, translational rates, protein modifications, and other posttranslational mechanisms [62, 63].

Of the 33 identified proteins with differential abundance, 26 (78%) did not have differential expression of their cognate mRNAs in the total and polysomal fractions (Figures 5(c) and 5(d)). These proteins may be undergoing strong posttranscriptional, translational, and/or posttranslational regulation. As mentioned above, there is no differential expression of their cognate mRNAs of the nine upregulated proteins. Some of these proteins are related to lipid metabolism or the adipogenic process.

For example, type 2 insulin-like growth factor receptor (IGF2R) is a protein (also called the cation-independent mannose-6-phosphate receptor) that modulates the tissue and circulating levels of IGF2 by internalization and targeting lysosomes for degradation. Insulin exposure increases the steady-state number of IGF2R, in addition to increasing the ligation, internalization, and degradation of IGF2 [64, 65]. These findings are curious due to the high levels of IGF2 expression in obese individuals with type 2 diabetes mellitus and because high doses of IGF2 increase *in vitro* differentiation and lipid accumulation [66, 67]. Furthermore, Igf2r-KO directly reduced brown adipogenesis and brown adipocyte survival [68]. To date, the exact mechanism of action of IGF2R in the adipogenic differentiation process and adipose tissue has been unknown. In this study, we demonstrated the expression of IGF2R at the protein level in differentiating cells since the receptor may have been directly induced by insulin exposure and may play a role in the control of lipid accumulation at the very beginning of hASC differentiation.

Another protein that was shown to be upregulated and which responds to insulin is eukaryotic translational initiation factor 6 (EIF6). This protein regulates the translation of specific mRNAs through the control of 60S availability [69]. Recently, it was shown that eIF6 controls glycolysis and fatty acid synthesis, in addition to influencing the translational activation of adipogenic transcription factors (*e.g*., C/EBP*β* and ATF4) and lipogenic enzymes in the adipogenic process of MSC [70].

The focal adhesion fermitin family member 2 protein (FERMT2), also known as Kindlin-2 protein, is expressed in multiple cell types (including MSC) and plays a critical role during early embryogenesis since global protein deletion results in peri-implantation lethality in mice. FERMT2 appears to have essential functions in the chondrogenesis, osteogenesis, and adipogenesis of MSC [71]. Recently, it was demonstrated that the expression of FERMT2 in hMSC undergoing osteogenic differentiation was markedly increased. However, protein levels were reduced during adipogenesis (after 14 days). The knockdown of FERMT2 in hMSC results in cells spontaneously differentiating into adipocytes and PPAR*γ* and aP2 expression increasing significantly [72, 73]. Despite that, transgenic mice with the deletion of FERMT2 in adipocytes possess severe lipodystrophy with drastically reduced adipose tissue mass. Protein loss suppressed adipocyte gene expression and differentiation [74]. In this study, we show only the differential abundance of the FERMT2 protein (not cognate mRNA expression) in early adipogenesis of hASC. This may indicate a transient expression throughout the differentiation process, and new studies should be conducted to better understand the gene's expression dynamics.

Emerin protein (EMD) is anchored in the inner nuclear membrane. It binds and regulates the nuclear accumulation of the *β*-catenin protein (Wnt signaling) [75]. In mouse preadipocytes undergoing adipogenesis, *β*-catenin was downregulated at the protein level and was accompanied by a significant upregulation of emerin (mRNA and protein level). Emerin controls the redistribution of *β*-catenin from the nucleus to the cytoplasm, facilitating its degradation and consequently allowing the adipogenic program [76]. Despite this, it has been shown that after 10 days of adipogenic differentiation, a fraction of adipocytes (approximately 70%) lacked emerin and other proteins related to nuclear lamina [77]. Our results, therefore, indicate that emerin may play a role in *β*-catenin regulation at the very beginning of adipogenesis in hASC, being subsequently regulated throughout the differentiation process.

Fibroblast growth factor (FGF) signaling is another important pathway that controls stem cells [78]. For example, FGF2 has the ability to inhibit the adipogenic process. The lysyl oxidase protein (LOX) catalyzes the cross-linking of lysine residues in elastin and collagen, fortifying the extracellular matrix. It partially enhances the adipogenesis of preadipocytes through the inhibition of FGF2 receptor signaling (including downregulation of AKT and ERK1/2) [79]. Interestingly, the TGF- *β*/SMAD signaling pathway plays a critical role in adipocyte commitment of MSCs as well. Both BMP4 and BMP2 activated the expression and phosphorylation of SMAD1/5/8 to form a complex with SMAD4. This complex is translocated to the nucleus to regulate LOX during adipocyte commitment [80, 81]. Here, we identified the expression of SMAD4 in 24 h-differentiated cells (Supplementary Table 1) and found that LOX was differentially abundant at the protein level in these induced cells (Supplementary Table 2). The remaining identified proteins (ATP1A1, ETHE1, PACSIN2, and BUB3) may play important roles in mitochondrial metabolism, lipid metabolism, vesicular traffic, cell migration, and cell cycle regulation [82–85].

On the other hand, we identified 17 downregulated proteins with no differential expression of their cognate mRNAs. These proteins may be undergoing extreme proteolytic activity since their abundance is critical for starting adipogenesis in hASC. Some of these proteins exhibit antiadipogenic activity, suppressing the differentiation process. This is the case of alpha-2-macroglobulin (A2M), a protease inhibitor protein. Accumulation of the A2M protein in murine preadipocytes inhibits adipogenesis, while the depletion of intracellular A2M increases lipid accumulation and adipocyte-gene marker expression [86]. Curiously, adult bovine serum does not support adipogenic differentiation in a similar manner to fetal bovine serum since the A2M concentration is 3.5 times higher [87].

Finally, the two most downregulated proteins identified here (QPRT and SDF4) have no known relationship with adipogenesis. Quinolinate phosphoribosyl transferase protein (QPRT) catabolizes quinolinic acid to nicotinic acid mononucleotide for de novo NAD synthesis (kynurenine pathway). It has been shown that both L-kynurenine and L-tryptophan significantly increase the stemness and migration of hBMSC, in addition to suppressing adipogenesis [88–90]. The rapid degradation of QPRT may thus be necessary for the loss of self-renewal capacity in hASC and triggering adipogenesis. The stromal cell-derived factor 4 protein (SDF-4), also called Cab45, is a Ca^2+^-binding protein involved in cell migration and proliferation through regulation of the cytosolic calcium level. Ca^2+^ plays an important role in different stages of hMSC differentiation and proliferation [91]. For example, hASC exposed to Ca^2+^ significantly reduced adipogenic differentiation and triglyceride content [92]. The SDF-4 downregulation we observed may therefore exert control on hASC proliferation through cytosolic Ca^2+^ regulation.

Taken together, these results represent novel information on the regulation of gene expression at the beginning of the adipogenic differentiation process in hASC. The processes that regulate mRNA transcription and translation and protein degradation are thus critical for human adipogenesis.

## 5. Conclusion

In conclusion, we have demonstrated that the initial step of the adipogenic differentiation process is highly controlled at the transcriptional, posttranscriptional, and posttranslational levels. We were able to identify proteins with potentially important roles in adipocyte commitment at the very beginning of differentiation. These results may contribute to a better understanding of human adipogenesis and the development of obesity.

## Figures and Tables

**Table 1:** Subjects characteristics.

Subjects	Donor 1	Donor 2	Donor 3	Mean ± SD
Age	46	20	48	38 ± 12.75
Gender	F	F	F	F
Weight (kg)	74.5	75	90	79.8 ± 7.19
Height (cm)	166	174	175	171 ± 4.02
BMI	27.04	24.77	29.39	27.06 ± 1.88

## Data Availability

The raw proteomic data used to support the findings of this study have been deposited in the PRIDE database under the accession number PXD026299. The raw RNA-seq data were downloaded from the ArrayExpress repository under the accession number E-MTAB-6298. The analyzed datasets used to support the findings of this study are included within the article.

## Supplementary Materials

Supplementary Materials Figure S1: human adipose-derived stem cell (hASC) characterization. Figure S2: sample chromatographies. Figure S3: Gene Ontology (GO) analysis of identified proteins in the proteomics assay. Figure S4: Gene Ontology (GO) analysis of differentially abundant proteins identified in the proteomic assay. Figure S5: mass spectrometer spectrum of identified hidden proteins in CT, ADI, and in both conditions. Table S1: proteins identified in the proteomic analysis of undifferentiated and induced hASC. Table S2: differentially abundant proteins during the first 24 hours of adipogenesis. Table S3: expression quantification of identified proteins and peptides. Table S4: differentially expressed genes in the total and polysomal RNA-seq during the first 24 hours of adipogenesis.
