# Supplementary material for: Proteogenomic Analysis Reveals Proteins Involved in the First Step of Adipogenesis in Human Adipose-Derived Stem Cells
Source: Stem Cells Int. 2021 Dec 16;2021:3168428. doi: 10.1155/2021/3168428 (PMC8702357; doi:10.1155/2021/3168428)
Supplement: Supplementary Materials — Figure S1: human adipose-derived stem cell (hASC) characterization. Figure S2: sample chromatographies. Figure S3: Gene Ontology (GO) analysis of identified proteins in the proteomics assay. Figure S4: Gene Ontology (GO) analysis of differentially abundant proteins identified in the proteomic assay. Figure S5: mass spectrometer spectrum of identified hidden proteins in CT, ADI, and in both conditions. Table S1: proteins identified in the proteomic analysis of undifferentiated and induced hASC. Table S2: differentially abundant proteins during the first 24 hours of adipogenesis. Table S3: expression quantification of identified proteins and peptides. Table S4: differentially expressed genes in the total and polysomal RNA-seq during the first 24 hours of adipogenesis. [file 3168428.f1.zip › Supplementary_Figures (2).docx]

**Supplementary Figures**

**From**

**Proteogenomics Analysis Reveals Proteins Involved in the First Step of Adipogenesis in Human Adipose-derived Stem Cells**

Bernardo Bonilauri^1^, Amanda C. Camillo-Andrade^2^, Marlon D. M. Santos^2,^ Juliana de S. da G. Fischer^2^, Paulo C. Carvalho^2^, Bruno Dallagiovanna^1^*

^1^ Laboratory of Basic Biology of Stem Cells (LABCET), Carlos Chagas Institute - Fiocruz/PR, Curitiba, Paraná, 81350-010, Brazil.

^2^ Laboratory for Structural and Computational Proteomics, Carlos Chagas Institute - Fiocruz/PR, Curitiba, Paraná, 81350-010, Brazil.

**SUPPLEMENTARY FIGURE S1:**

**Figure S1**. **Human adipose-derived stem cells (hASC) characterization.** **(A)** Representative flow citometry histograms for the corresponding cell-surface antigens: positive markers (CD90, CD105 and CD73 - upper panels) and, negative markers (CD34, CD45 and HLA-DR - lower panels). **(B)** Immunofluorescence showing non-induced hASC (Control) and adipogenic induced cells (Induced) for 14 days. Lipid droplets were stained green with Nile Red stain. Bar plot showing adipogenic differentiation quantification after 14 days of differentiation (n=3). *** p < 0,001.

**SUPPLEMENTARY FIGURE S2:**

**
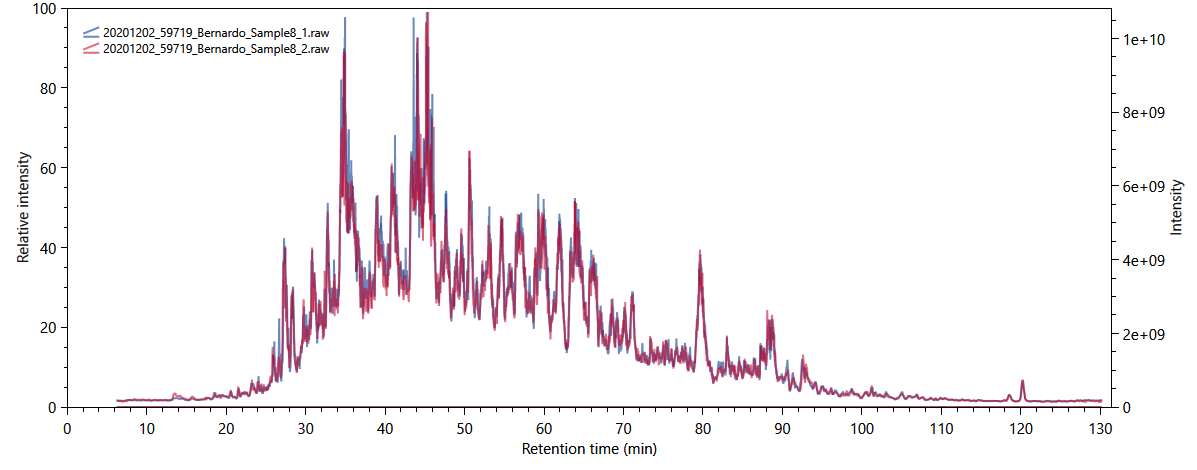
**

**
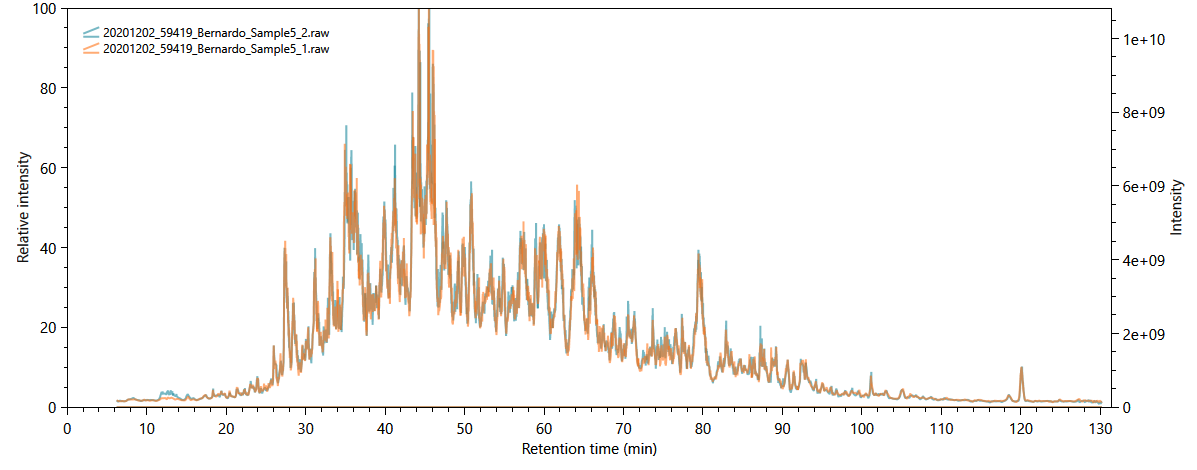
**

**Figure S2**. **Samples chromatographies**. Chromatography comparison of ADI technical replicates (upper panel) and comparison of CT technical replicates (lower panel).

**SUPPLEMENTARY FIGURE S3:**

**Figure S3:** **Gene Ontology (GO) analysis of identified proteins in the proteomics assay**. **(A)** Biological processes related to common identified proteins. **(B)** Biological processes related to CT unique identified proteins.

**SUPPLEMENTARY FIGURE S4:**

**Figure S4:** **Gene Ontology (GO) analysis of differentially abundant proteins identified in the proteomic assay.** **(A)** Biological processes related upregulated proteins after adipogenic differentiation triggering (24h). **(B)** Biological processes related downregulated proteins after adipogenic differentiation triggering (24h).

**SUPPLEMENTARY FIGURE S5:**

- P04114 – SLVTTLNSDLK - 2.555 - CT UNIQUE


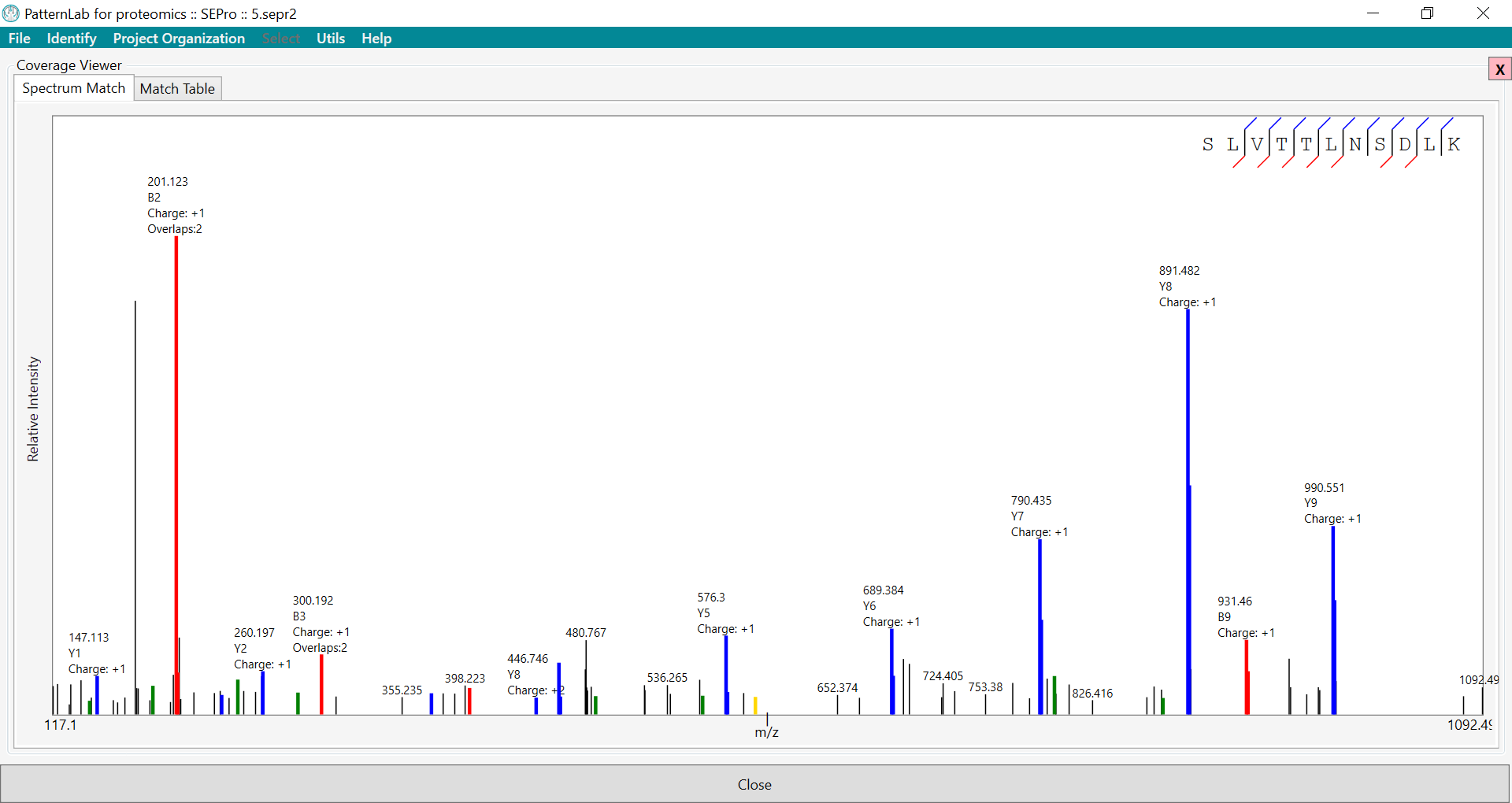


- P03915 – TISQHQISTSIITSTQK - 2.6791 - 2.6791 - CT UNIQUE


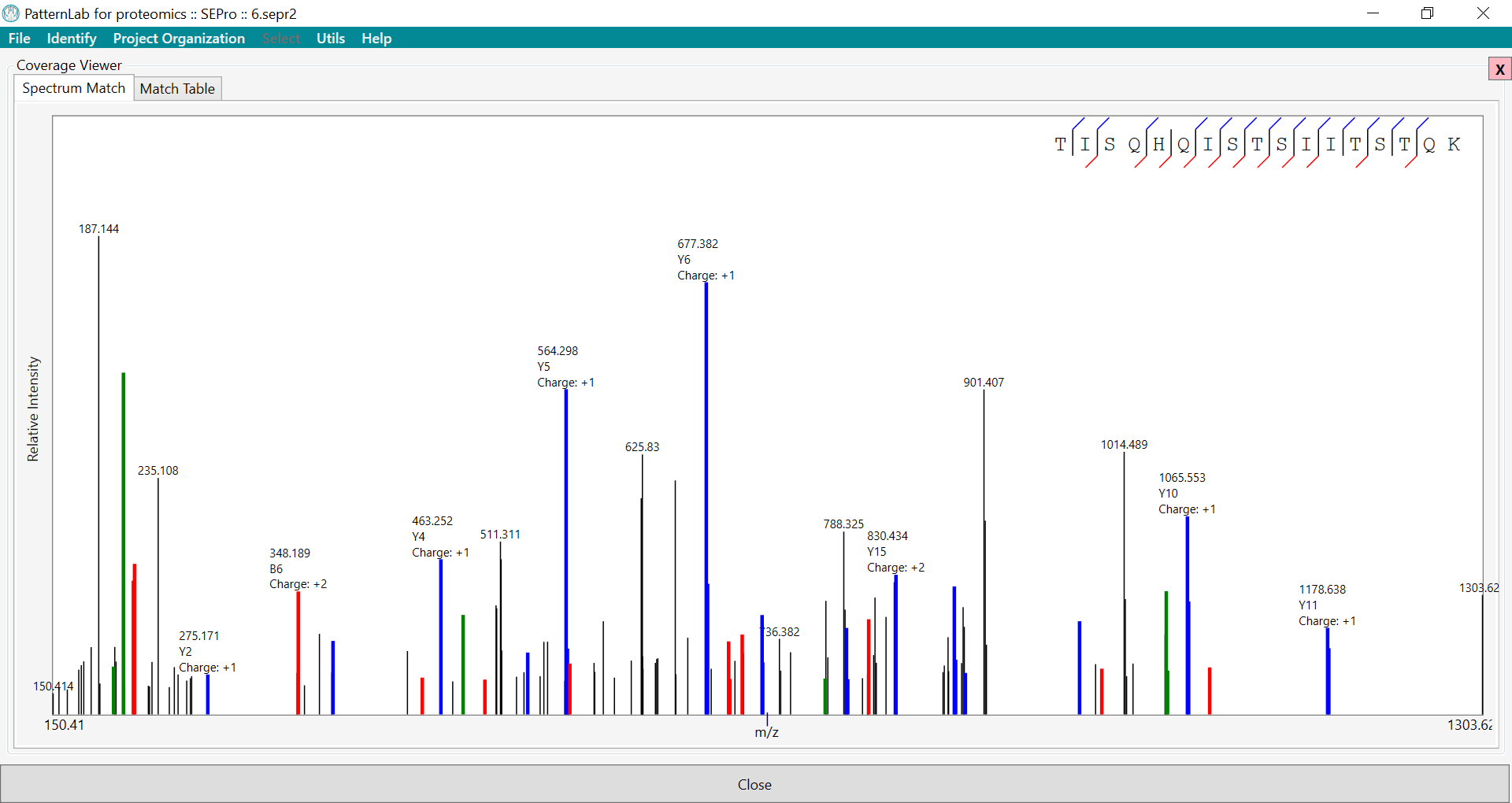


- O95182 – ALVSGKPAESSAVAATEK - 3.5844 - ADI UNIQUE


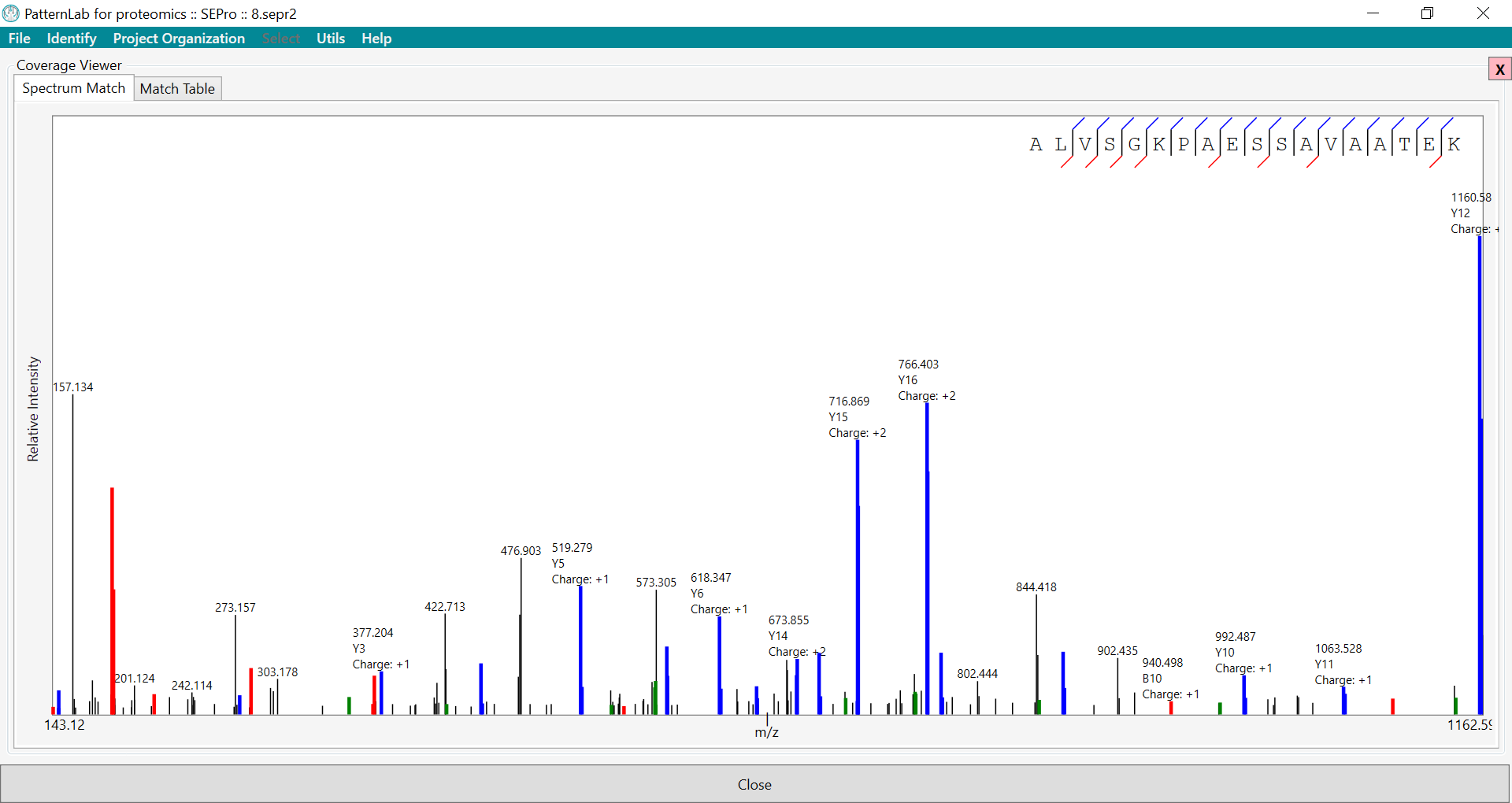


- P81605 – YDPEAASAPGSGNPCHEASAAQK - 4.3124 - ADI UNIQUE


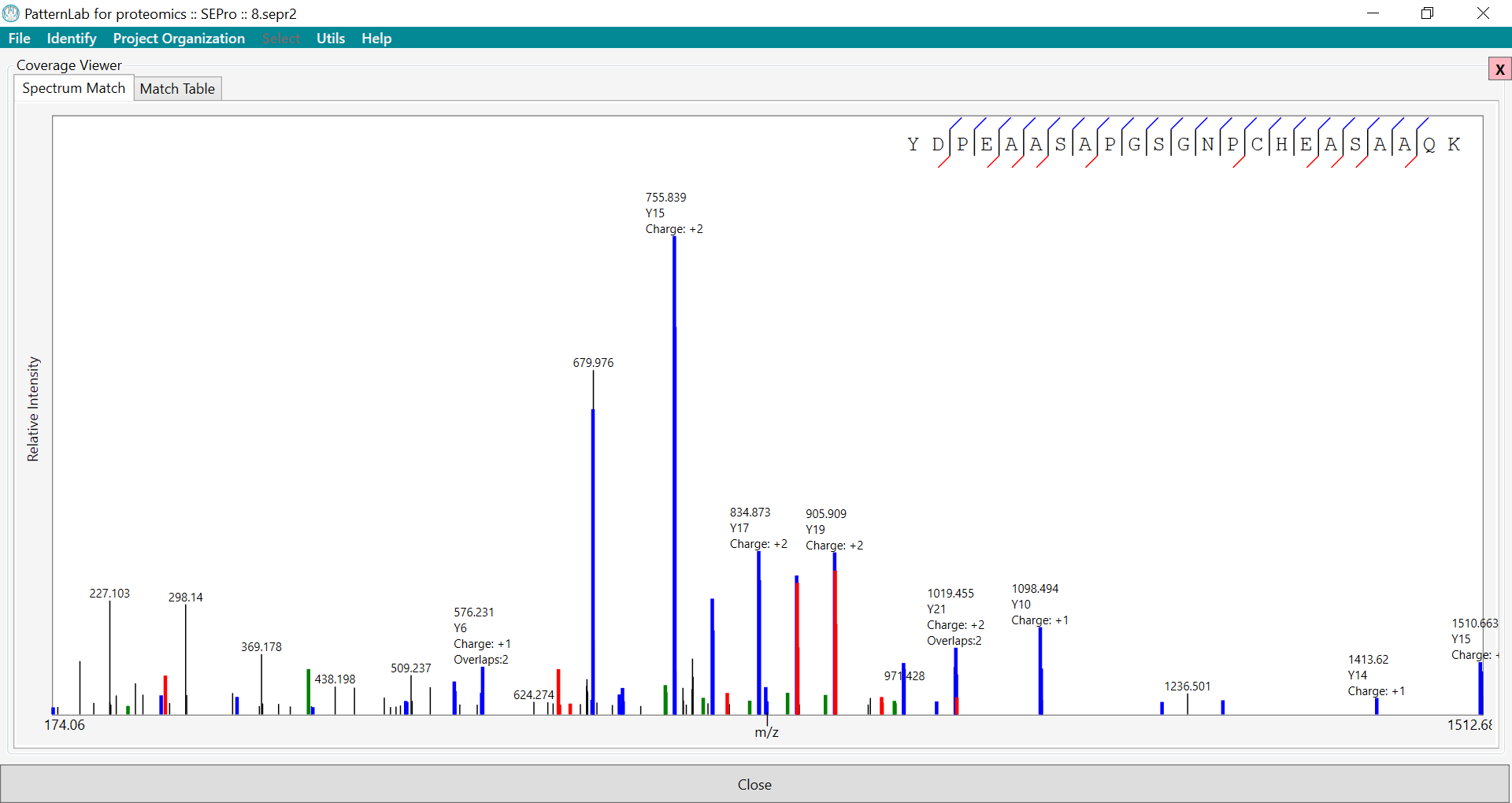


- P02768 – DVFLGMFLYEYAR – 2.5854 - BOTH


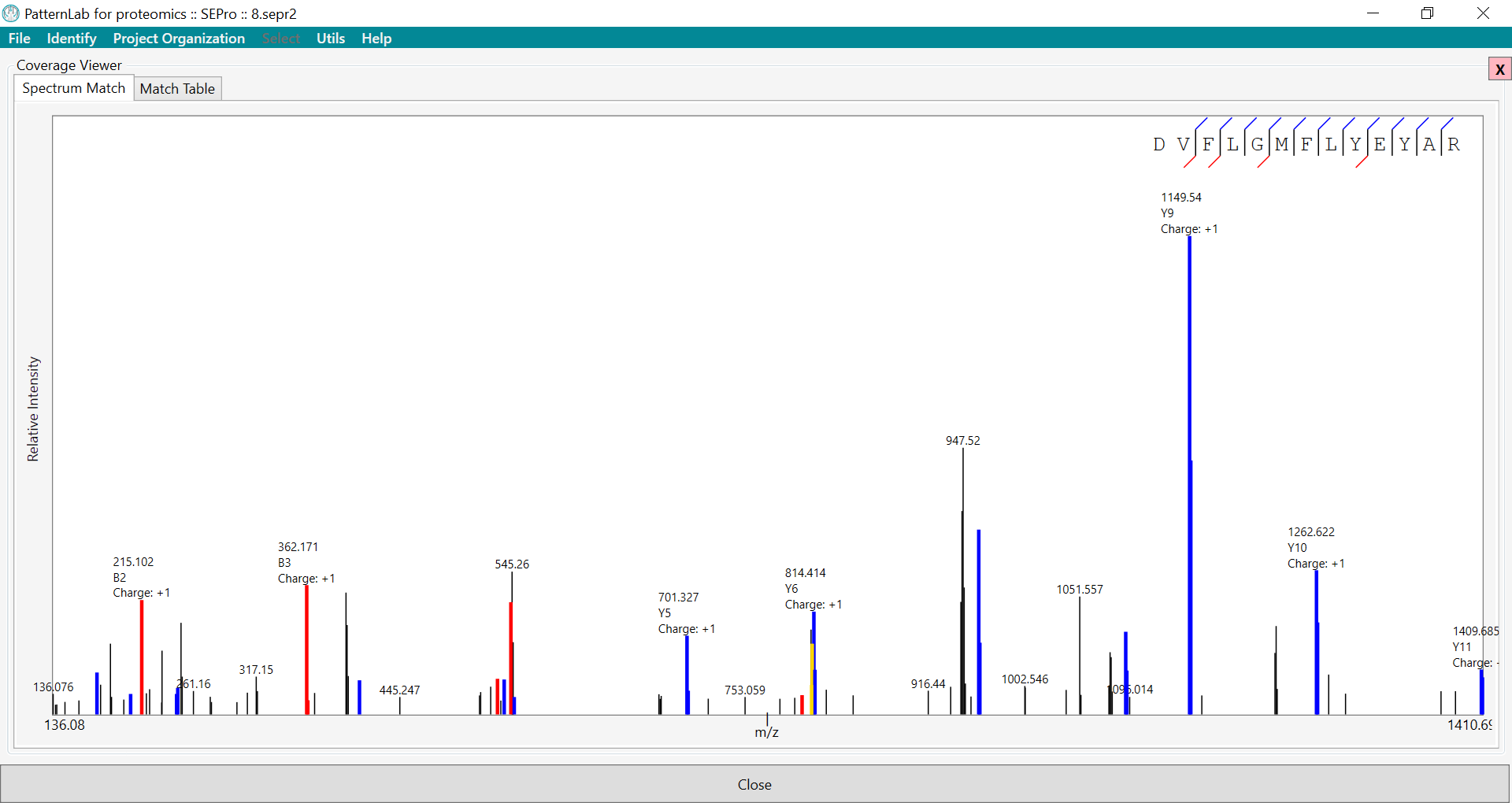


- P12821 – TATCWSLDPDLTNILASSR – 3.8061 - BOTH


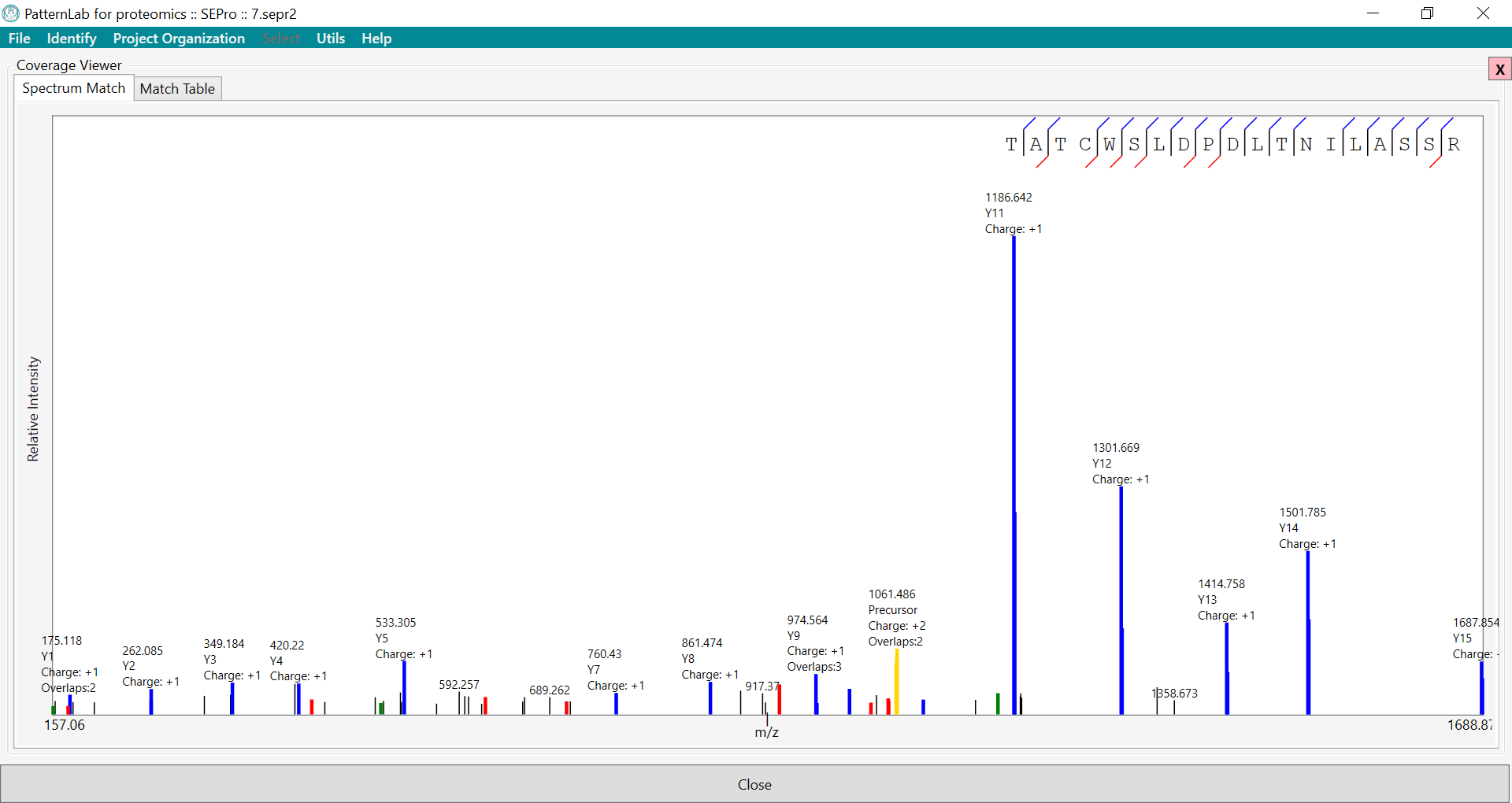


- P69905 – TYFPHFDLSHGSAQVK - 4.0586 - BOTH


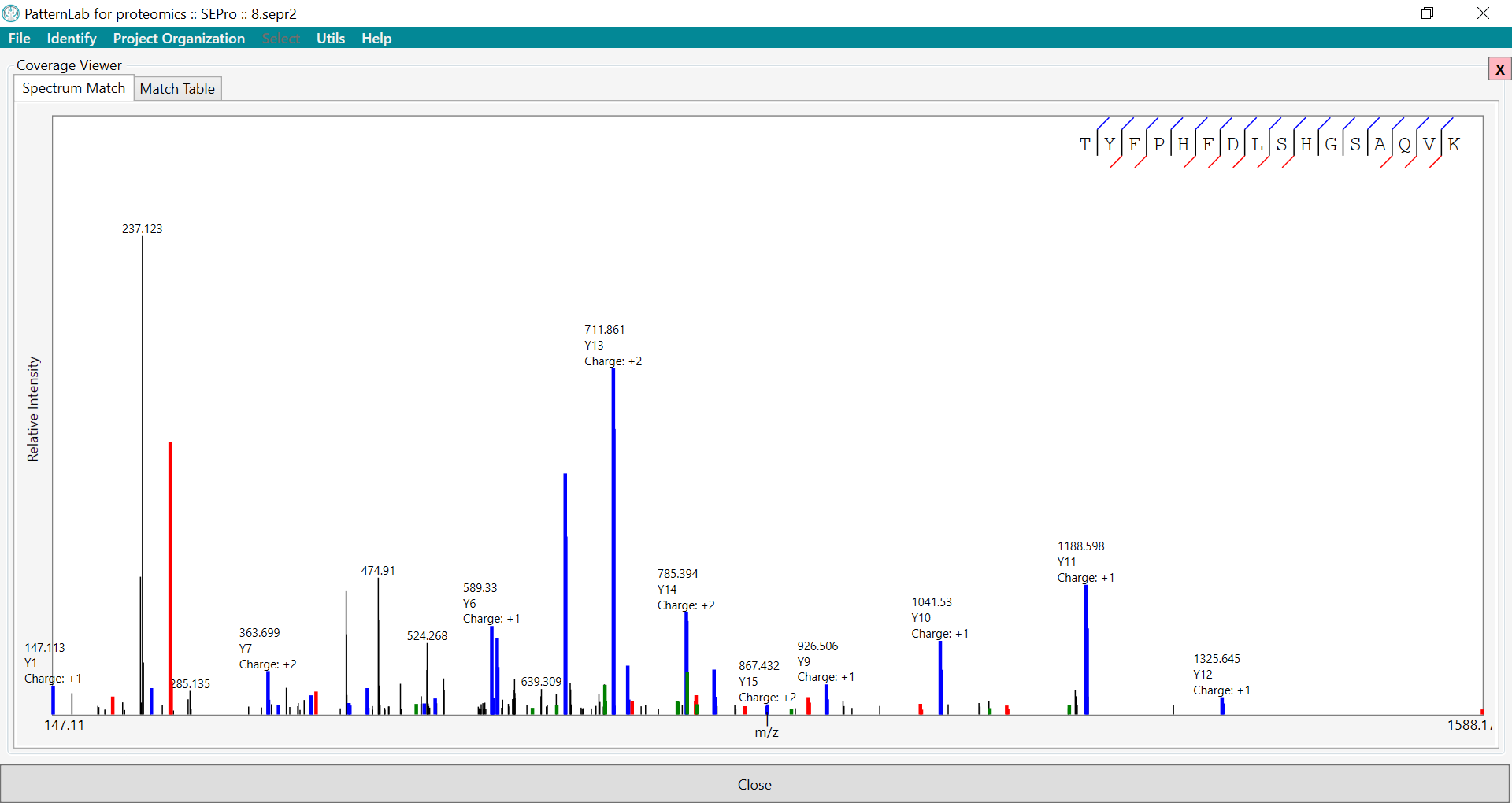


- P14174 – PMFIVNTNVPR – 3.0573 - BOTH


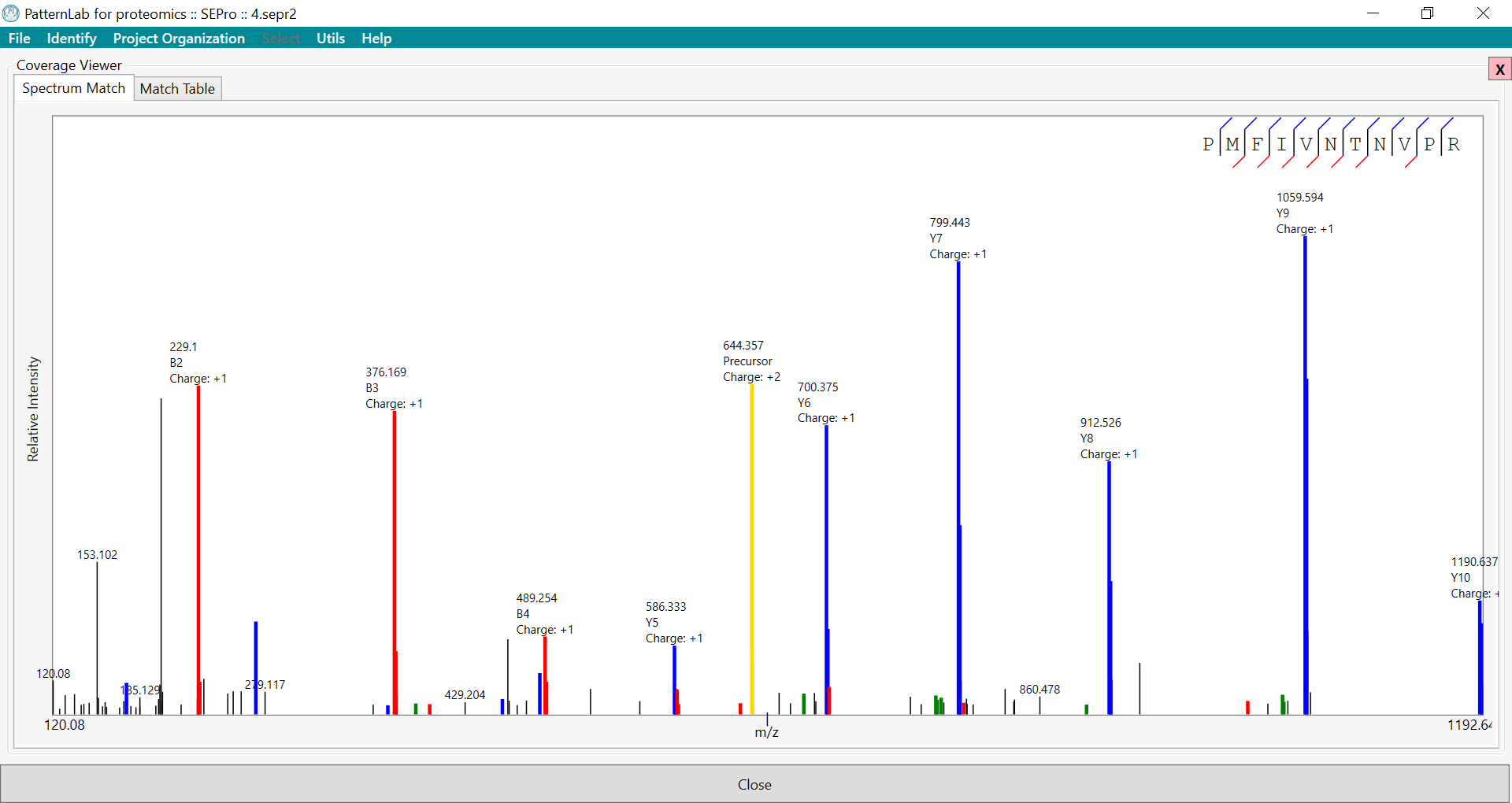


**Figure S5:** Mass spectrometer spectrum of identified hidden proteins in CT, ADI and in both conditions. Images titles refer to protein ID, sequence, spectrum score and condition, respectively.
